# Investigating structural and optoelectronic properties of Cr-substituted ZnSe semiconductors

**DOI:** 10.1038/s41598-024-66378-2

**Published:** 2024-07-05

**Authors:** Muhammad Aamir Iqbal, Sunila Bakhsh, Siti Sarah Maidin, Kareem Morsy, Jeong Ryeol Choi, Arnold C. Alguno

**Affiliations:** 1 School of Materials Science and Engineering, Zhejiang University, Hangzhou, 310027 China; 2 Department of Physics, Balochistan University of Information Technology, Engineering and Management Sciences, Quetta, 87300 Pakistan; 3 Faculty of Data Science and Information Technology, INTI International University, 71800 Nilai, Malaysia; 4 Biology Department, College of Science, King Khalid University, 61421 Abha, Saudi Arabia; 5 School of Electronic Engineering, Kyonggi University, Suwon, Gyeonggi-do 16227 Republic of Korea; 6 Department of Physics, MSU-Iligan Institute of Technology, 9200 Iligan City, Philippines; 7 Research Centre for Energy Efficient Materials, Premier Research Institute of Science and Mathematics, MSU-Iligan Institute of Technology, 9200 Iligan City, Philippines

**Keywords:** Cr-substituted ZnSe semiconductors, Cubic symmetry, Concentration dependency, Bandgap, Density of states, Infrared absorbance, Optoelectronics, Condensed-matter physics, Materials for devices, Structural materials, Theory and computation, Materials science, Physics

## Abstract

The optoelectronic and structural characteristics of the Zn_1−x_Cr_x_Se (0 ≤ x ≤ 1) semiconductor are reported by employing density functional theory (DFT) within the mBJ potential. The findings revealed that the lattice constant decreases with increasing Cr concentration, although the bulk modulus exhibits the opposite trend. ZnSe is a direct bandgap material; however, a change from direct to indirect electronic bandgap has been seen with Cr presence. This transition is caused by structural alterations by Cr and defects forming, which results in novel optical features, including electronic transitions. The electronic bandgap decreases from 2.769 to 0.216 eV, allowing phonons to participate and improving optical absorption. A higher concentration of Cr boosts infrared absorption and these Cr-based ZnSe (ZnCrSe) semiconductors also cover a wider spectrum in the visible range from red to blue light. Important optical parameters such as reflectance, optical conductivity, optical bandgap, extinction coefficient, refractive index, magnetization factor, and energy loss function are discussed, providing a theoretical understanding of the diverse applications of ZnCrSe semiconductors in photonic and optoelectronic devices.

## Introduction

The development of semiconductor devices leads to exceptional advances in various electronic and photonic technologies, such as information storage, signal processing, and visible display, which challenge the engineering of optoelectronic devices in terms of both cost and technology^1^. Considering this, the semiconductor materials of groups II-VI have significant and unique features that may be tuned by modifying the composition of the constituent compounds, resulting in tuned electronic and optical responses that are crucial for photoelectronic devices for commercial purposes and operate over a broad variety of wavelength spectra^2,3^. The direct and indirect electronic bandgap properties of the II–VI semiconductor materials involving transition metal-doped ZnSe alloys can make them an attractive candidate for various industrial applications in optoelectronic, photonic, and magnetic sensing devices^1–8^. ZnSe is one of the first II–VI semiconductors discovered with exceptional electronic and optical properties, having prominent applications in linear and nonlinear optics, flat panel displays, light-emitting diodes, lasers, and logic gates. Its two commonly known allotropes are cubic zinc-blende and hexagonal wurtzite structures, and bulk ZnSe has a wide direct bandgap of approximately 2.70 eV^8^. Such direct bandgap characteristics is essential and ideal for optoelectronic, photonic, and infrared optical devices^9,10^.

ZnCrSe materials have been widely explored in the existing literature for a variety of applications, including infrared lasers, spintronics, and luminescence^8–15^. Wei et al. examined the optical spectra of Cr-doped ZnSe structures utilizing a unique synthesis process that demonstrated good light-absorption characteristics in the 1800–3000 nm wavelength range, which corresponds to mid-infrared^13^. This considerably improves the material's efficiency, which is critical for high-power laser applications. The photoluminescence analysis of distinct emissions around the bandgap energy levels in ZnSe has proven to be an effective method to assess the effects of dopants and impurities, along with the accompanying transitions. Typically, ZnCrSe materials are reported to emit mid-infrared coherent light efficiently. Thus, they have better emission efficiency and tunability over mid-infrared wavelengths^14^. This characteristic is crucial for achieving high output power and enabling efficient pumping by common laser diodes. In addition to this, they possess high thermal stability, making them appropriate for high-power laser applications in spectroscopy, surgery, and other fields^15^. Several theoretical studies were performed to analyze the structural, optical, and electrical properties of transition metal-doped chalcogenide materials^4,7,16–19^. Zhang and his team reported a first-principles analysis of the absorption and luminescence properties of Cr^2+^-doped ZnSe crystals^17^. They also noted the size-dependent optical and electrical characteristics of ZnCrSe nanowires and suggested a decrease in electronic bandgap with the increase in size^18^. Recently, DFT simulations have been utilized to examine the electrical and optical properties of ZnCrSe nanosheets, indicating that doping induces the formation of a large number of defect bands in the center of the intrinsic bandgap. These intrinsic bandgaps are predominantly made up of Cr-d orbital electrons, Se-p orbital electrons, and d-p hybridized bands. The doping of Cr in ZnSe also alters the distribution of LUMO and HOMO levels. The HOMO–LUMO shift caused by Cr doping appears to boost the performance and adaptability of ZnSe in mid-infrared laser applications^19^.

The optoelectronic and structural characteristics of the ZnCrSe semiconductors are important in defining some significant features of the material, such as electron-photon interactions and inter-atomic forces, and identifying the optical and transport coefficients. The present study is aimed at modifying the existing state of the materials by combining electronic and optical parameters using an all-electron approach. In this study, we investigated the optoelectronic and structural properties of the ZnCrSe semiconductors using DFT calculations involving generalized gradient approximation (GGA) functional and modified Becke and Johnson (mBJ) potential to identify the potentiality of the use of these materials in electronic and photonic devices. This work reports significant and thorough attributes of ZnCrSe semiconductors and also directs the future applicability of these findings in the industry with further investigations, encouraging experimentalists to pursue commercial applications of these cubic-symmetric alloy materials in optoelectronics.

## Theoretical method

The full-potential linearized augmented plane-wave (FP-LAPW) method was used to solve the Kohn–Sham equations^20^ within the framework of DFT^21^, which was programmed in the Wien2k code^22^. The FP-LAPW method yields the eigenvalues by treating the interstitial regions and muffin-tin spheres. The Fourier series characterizes basis functions; however, the Schrödinger wave equation was used to describe the potential's spherical component^23^. The value of L_max_ = 10 was applied to the spherical harmonics of the muffin-tin spheres, and the G_max_ value was set at 12 by taking R_MT_ × K_max_ = 7.0. The energy separating the core and valence states was computed as − 7.0 Ry, while a denser mesh of k-points of 2000 (12 × 12 × 12) in the irreducible Brillion Zone was used. The convergence criteria for energy, charge, and force of the system were set to 0.00001 Ry, 0.0001 e and 1 mRy/Bohr, respectively. GGA^24^ was employed as an exchange–correlation functional for structural optimization, and mBJ-GGA^25^ was used to determine the Cr content-dependent electronic band structures and optical spectra.

## Results and discussion

**Figure 1:**
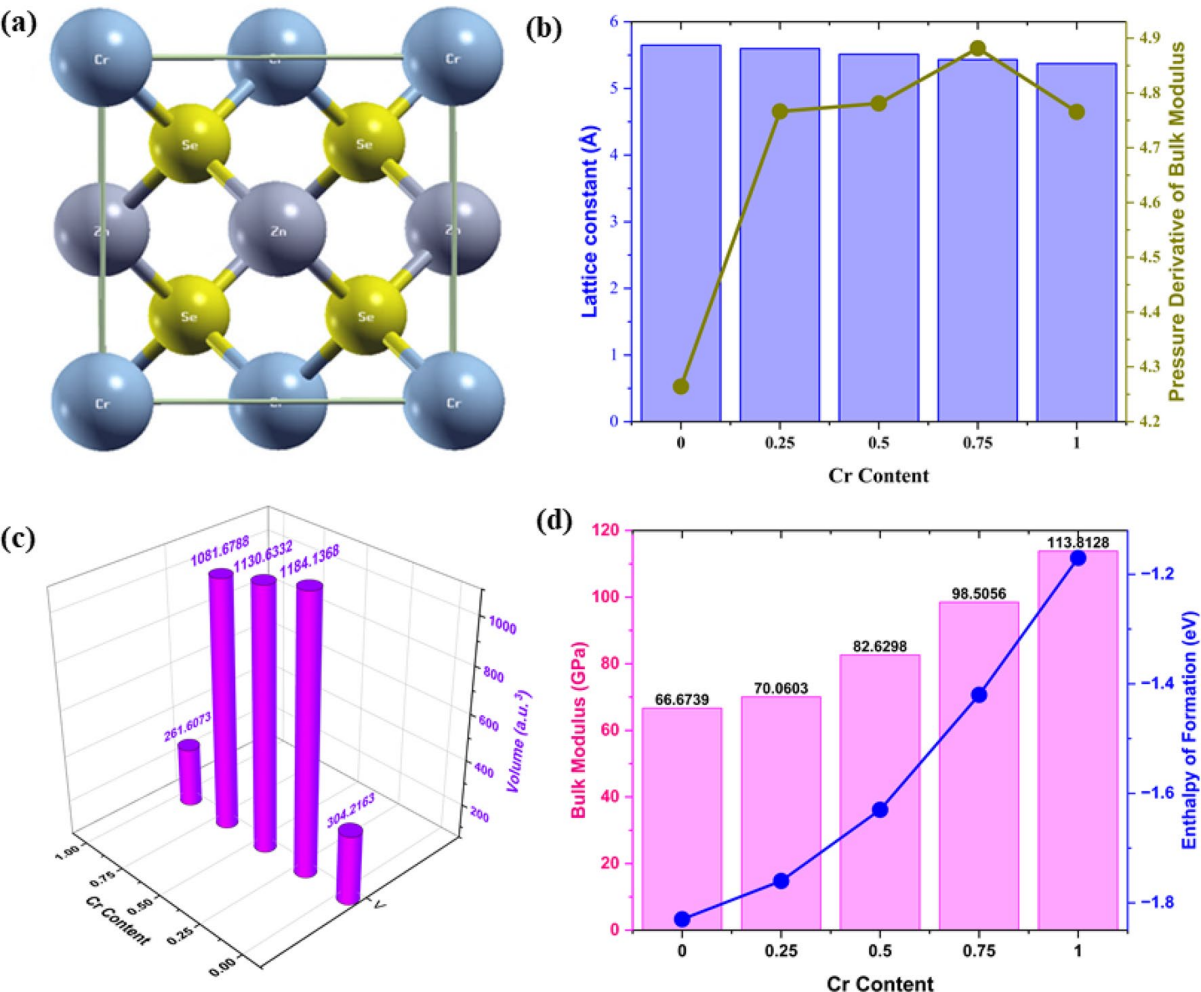
Cr concentration-dependent structural parameters. (**a**) Crystal structure of Cr_0.50_Zn_0.50_Se alloy, (**b**) Change in the pressure derivative of bulk modulus and lattice constant, (**c**) Change in volume, and (**d**) Change in bulk modulus and enthalpy of formation.

To explore the significant properties of a crystal, the optimized lattice parameters can be computed at an equilibrium state with a minimum correlating ground state energy. For this, we used the Birch-Murnaghan equation of state^26^, through which the obtained structural parameters with the incorporation of Cr at varying concentrations are displayed in Fig. 1. The associated optimization plots of minimum energy and volume are displayed in Figure S1 in Supplementary Material. To investigate the impact of Cr substitution, the structural and optoelectronic properties of ZnCrSe semiconductors were exploited by generating a unit cell of 1 × 1 × 1 dimension as reported earlier for Co-doped ZnSe^4^. All these generated structures were analyzed in cubic symmetry at varying concentrations of Cr (0 ≤ x ≤ 1). Figure 1a shows the ZnCrSe cubic crystal structure at a Cr concentration of 0.50, from which it can be seen that half of the Zn atom sites were substituted with Cr atoms. The difference in ionic radii of Cr and Zn leads to a decrease in the lattice constant and is related to the high concentration of Cr, whose atoms substitute Zn sites in the supercell of ZnCrSe alloys. This may also result in a decrease in the bond lengths, resulting in a small contraction in the lattice. The decreasing trend in the lattice constant is shown in Fig. 1b, which almost follows Vegard’s law stating that the lattice constant of an alloy’s crystal structure has a linear relationship with its elemental composition^27^. To establish the stability of ternary alloys, we estimated the enthalpy of formation (Δ*H*) utilizing the energy difference between the ternary alloys and their atoms using the method reported in the literature^4,28^. For this, we employed the relationship Δ*H* = *E*_tot_ (Zn_x_Cr_y_Se_z_) – x*E*_Zn_ – y*E*_Cr_ – z*E*_Se_, where x, y, and z depict the number of atoms, while *E*_Zn_, *E*_Cr_, and *E*_Se_ are the total energies of Zn, Cr, and Se atoms in a unit cell and *E*_tot_ (Zn_x_Cr_y_Se_z_) represents the total energy of the alloyed system. The stability is confirmed by the negative values of Δ*H* and our results agree with the reported literature^4^. Figure 1c portrays volume variation with the presence of Cr, while Fig. 1d shows the bulk modulus variation and enthalpy of formation with increasing Cr concentration.

All the structural parameters computed at varying Cr concentrations, including lattice constant, optimized minimum energy, and bulk modulus of the material, are summarized in Table S1. The analysis of parameters depicts the cubic structure of the investigated alloys at all Cr concentrations, specifically at 0.50, which is a unique and novel improvement in the existing computational literature and agrees with the past findings of transition metal-doped ZnSe^2,4,6,29–31^. This study shows that the bulk modulus of ZnCrSe increases with the increasing concentration of Cr because the toughness of the material increases, indicating CrSe has a stronger and larger bulk modulus than ZnCrSe alloys and ZnSe, depicting much less compressibility in comparison with its counterparts. This analysis shows that ZnCrSe semiconductors with high Cr content can be potentially used in high-pressure technological devices where the device’s stability is crucial^6^.

**Figure 2:**
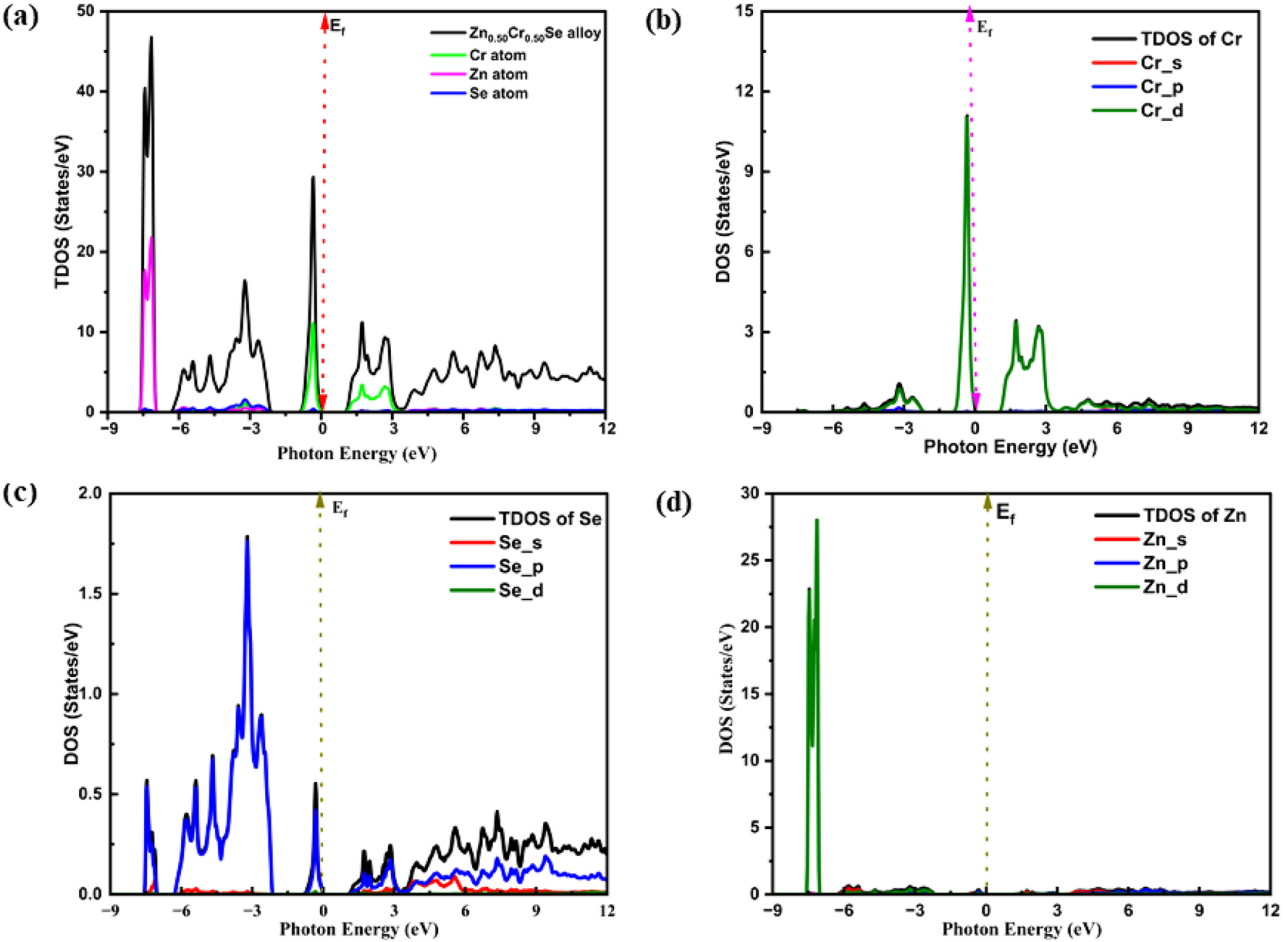
Change in total and partial density of states of Zn_0.50_Cr_0.50_Se alloy. (**a**) Zn_0.50_Cr_0.50_Se alloy, (**b**) Cr atom, (**c**) Se atom, and (**d**) Zn atom.

**Figure 3:**
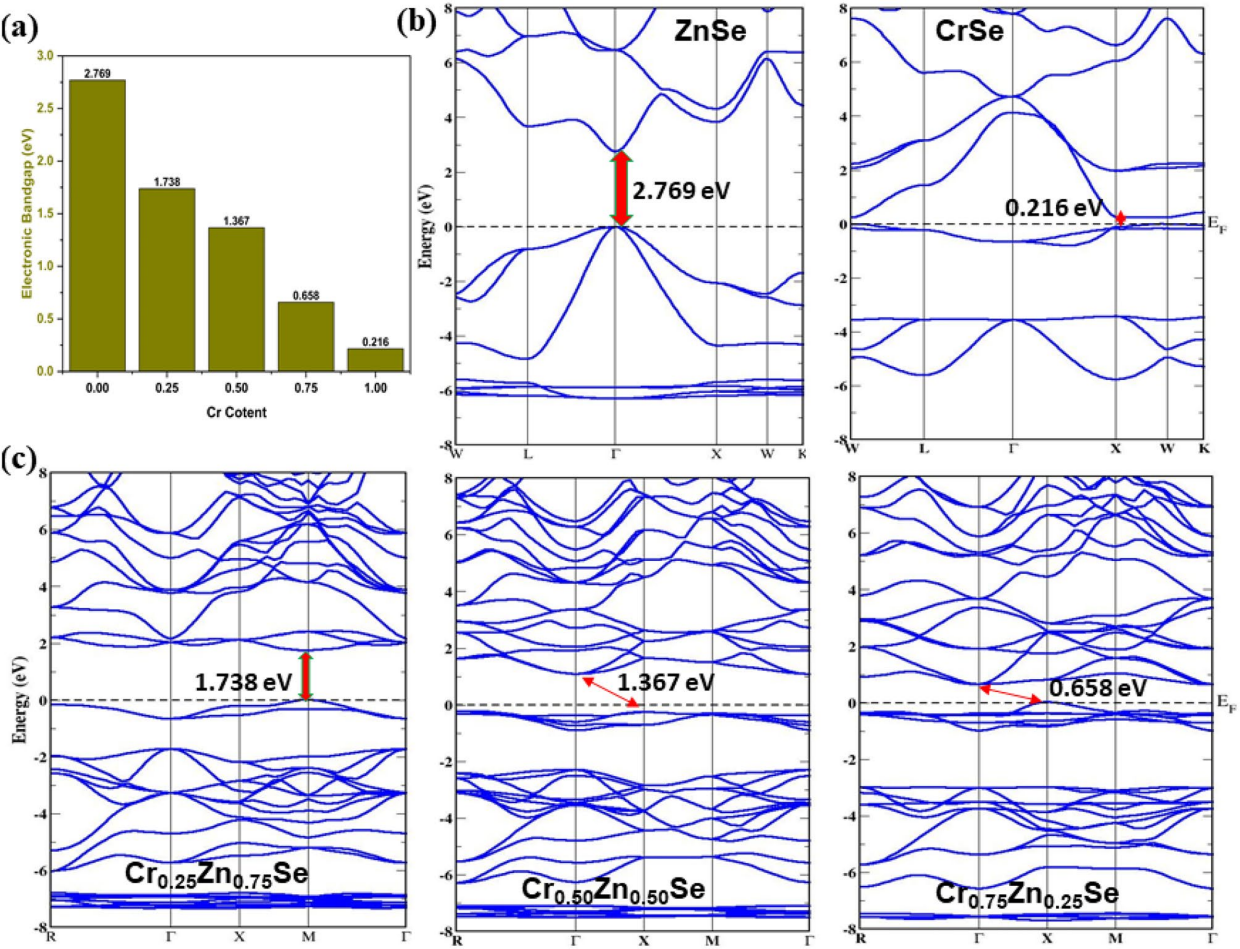
Cr content-dependent electronic bandgap and band structures of ZnCrSe materials. (**a**) Change in the electronic bandgap, (**b**) Band structures of ZnSe and CrSe, and (**c**) Band structures of Cr_0.25_Zn_0.75_Se, Cr_0.50_Zn_0.50_Se, and Cr_0.75_Zn_0.25_Se.

The density of states (DOS) and electronic properties of the ZnCrSe semiconductors were investigated at the first Brillouin zone, where the symmetry points were high. The total DOS (TDOS) at varying Cr content for all semiconductors is displayed in Figure S2 in the photon energy range of − 8 to 8 eV and TDOS along with partial DOS (PDOS) of Zn_0.50_Cr_0.50_Se alloy in the energy range of − 9 to 12 eV is displayed in Fig. 2. The TDOS and PDOS were investigated deeply to clearly understand the electronic behavior and orbital origins in the ZnCrSe semiconductors at varying Cr content and the results show that the DOS of these materials is largely influenced by Cr content. The incorporation of Cr introduces impurity levels and defect states, which alter the electronic structure of ZnSe, giving birth to new electronic states. Figure S2 shows the change in TDOS as a function of Cr content, from which it can be seen that the higher DOS corresponds to 25 percent content of Cr. New electronic states can also be seen in the low-energy region of the valence band between the energy ranges of − 1.90 and − 7.95 eV. Also, a shift towards a low-energy region with Cr content can be observed in the conduction band, where DOS is significantly influenced by Cr content. To predict the characteristics of the Cr-influenced DOS, the TDOS along with the PDOS and atomic state contribution of the Zn_0.50_Cr_0.50_Se alloy are shown in Fig. 2. From this figure, it can be seen that the valence band is primarily composed of three regions, in which the first region near the fermi energy is composed of Cr-d along with the hybridization of Se-p and the minor contribution of d states. The second region is dominated by the Se-p and Cr-d states, with a minor contribution from the Cr-p and Zn-s states. The third region is contributed by the Zn-d and Se-p states along with minor addition of Se-s and Cr-d states. In general, the Cr presence shifted the valence band states down toward the low-energy region, as reported for Co doped ZnSe materials^4^. On the other side, the conduction band is mainly composed of Cr-d, Se-p, and s states, with a very minor contribution from the Zn-s and p states. In general, the origin of states in this band shifts towards the fermi level, depicting a decrease in the electronic bandgap as the generated band structures (see Fig. 3).

Analysis of electronic band structures shows a decrease in its electronic bandgap energy with increasing the concentration of Cr. The direct bandgap nature of a material can be determined by the maxima and minima locations of valence and conduction bands. If they align in the same k-space region, the material is taken as a direct bandgap and indirect otherwise. Here, ZnSe, CrSe, and Zn_0.75_Cr_0.25_Se are identified as direct bandgap materials, while others are indirect as X-Γ symmetry is observed (see Fig. 3b,c). The inclusion of Cr in ZnSe significantly modifies its electronic bandgap (see Fig. 3a), with a direct-to-indirect bandgap transition taking place at particularly important Cr concentrations of 50% and 75%. This effect results from the creation of Cr-induced impurity levels and defect states, which modify the electronic structure of ZnSe because Cr (3d^5^ 4s^1^) has a different electronic configuration than Zn (3d^10^ 4s^2^). Furthermore, the ionic radii of Cr differ from those of Zn, thus swapping Zn with Cr atoms alters crystal symmetry, causing distortions that might affect band topologies^32^. Also, the Cr substitution at Zn sites originates novel, size-dependent electronic transitions^33^. As Cr concentration increases, impurity levels and defect states alter the bandgap nature, causing a change from the direct to the indirect bandgap. This transition is accompanied by changes in optical characteristics such as absorption and reflection that are governed by Cr concentration. The observed bandgap adjustment has significant implications for the design of innovative optoelectronic devices and sensors that need customizable electrical and optical features^34,35^. Furthermore, this work demonstrates Cr doping's potential as a feasible way to tailor ZnSe's electronic bandgap, allowing for the development of novel device features and applications^36^. Similar effects have been reported in other semiconductor materials, including BeSe^16^, ZnTe^37^, and GaAs^38^, where transition metal doping causes considerable changes in electronic bandgap and optical characteristics^7^. The electronic bandgap energy as a function of Cr content is computed as 2.769 to 0.216 eV, as summarized in Table 1.

**Table 1:** Cr content-dependent bandgap energy of ZnCrSe semiconductors.

Semiconductor	Electronic bandgap (eV)	
	Present work	Literature
ZnSe	2.769	2.83^4^
		2.70^9^
		3.50^11^
		2.58^12^
		2.77^39^
		2.82^40^
		1.31^41^
Cr_0.25_Zn_0.75_Se	1.738	–
Cr_0.50_Zn_0.50_Se	1.367	–
Cr_0.75_Zn_0.25_Se	0.658	–
CrSe	0.216	–

The radiation-matter interaction is of great importance in analyzing the optical response of a material. It yields optical spectra, which are associated with the shifts in the electron probability from an unoccupied to an occupied state. This linear response of the system can be studied by the complex dielectric function, as well as joint densities, which is mathematically expressed as^42^:

1 $$\varepsilon \left(\omega \right)={\varepsilon }_{1}\left(\omega \right)+i{\varepsilon }_{2}\left(\omega \right)$$

Here, ${\varepsilon }_{2}\left(\omega \right)$ and ${\varepsilon }_{1}\left(\omega \right)$ present the imaginary and real parts of the dielectric function, respectively. The real part classifies the material’s polarization and presents the dielectric function at zero frequency. The imaginary part describes the absorbance and both of these can be computed using the Kramers–Kronig relationships of the forms^43^.

2 $${\varepsilon }_{1}\left(\omega \right)=1+\frac{2}{\pi }P{\int }_{0}^{\infty }\frac{{\omega }^{/} {\varepsilon }_{2}\left({\omega }^{/}\right)}{{\omega }^{/2}-{\omega }^{2}}d{\omega }^{/}$$

3 $${\varepsilon }_{2}\left(\omega \right)=-\frac{2\omega }{\pi }P{\int }_{0}^{\infty }\frac{({\varepsilon }_{1}({\omega }^{/})-1)}{{\omega }^{/2}-{\omega }^{2}}d{\omega }^{/}$$

where *P* denotes the Cauchy principal value of the integral and $\omega$^/^ shows the collective DOS.

The dielectric function can also be used to compute other optical parameters, such as extinction coefficient $(k\left(\omega \right))$ and refractive index $(n\left(\omega \right))$. $n\left(\omega \right)$ provides information about the decrease in the speed of incoming photons in any medium as a function of frequency^44^. Both the $n\left(\omega \right)$ and $k\left(\omega \right)$ can be mathematically expressed as:

4 $$n\left( \omega \right) = \frac{1}{\sqrt 2 }[\sqrt {\{ \mathop {\varepsilon_{1} }\nolimits^{2} \left( \omega \right) + \mathop {\varepsilon_{2} }\nolimits^{2} \left( \omega \right)\} } + \varepsilon_{1} \left( \omega \right)]^{1/2}$$

5 $$k\left( \omega \right) = \frac{1}{\sqrt 2 }[\sqrt {\{ \mathop {\varepsilon_{1} }\nolimits^{2} \left( \omega \right) + \mathop {\varepsilon_{2} }\nolimits^{2} \left( \omega \right)\} } - \varepsilon_{1} \left( \omega \right)]^{1/2}$$

The optical absorption of the material determines the amount of energy that a substance can absorb^44^. It depends on the imaginary part of the dielectric function and can be computed by analyzing the behavior of the extinction coefficient. Its mathematical formula is given by

6 $$\alpha \left( \omega \right) = \frac{4\pi k(\omega )}{{\mathop \lambda \nolimits_{0} }} = \frac{\omega }{nc}\varepsilon_{2} \left( \omega \right)$$

The response of a material surface can be studied by analyzing its reflectance spectra, which are generated using the relationship^44^.

7 $$R\left(\omega \right)=\frac{{\left(n\left(\omega \right)-1\right)}^{2}+{k}^{2}\left(\omega \right)}{{\left(n\left(\omega \right)+1\right)}^{2}+{k}^{2}\left(\omega \right)}$$

On the other hand, the real component of optical conductivity and energy loss function of interacting electrons can be determined from the dielectric function^44^:

8 $${Re}\sigma (\omega )=\frac{\omega }{4\pi }{\varepsilon }_{2}(\omega )$$

9 $$L\left(\omega \right)=\text{Im}\left(-\frac{1}{\varepsilon \left(\omega \right)}\right)=\frac{{\varepsilon }_{2}(\omega )}{\left({\varepsilon }_{1}^{2}\left(\omega \right)+{\varepsilon }_{2}^{2}\left(\omega \right)\right)}$$

**Figure 4:**
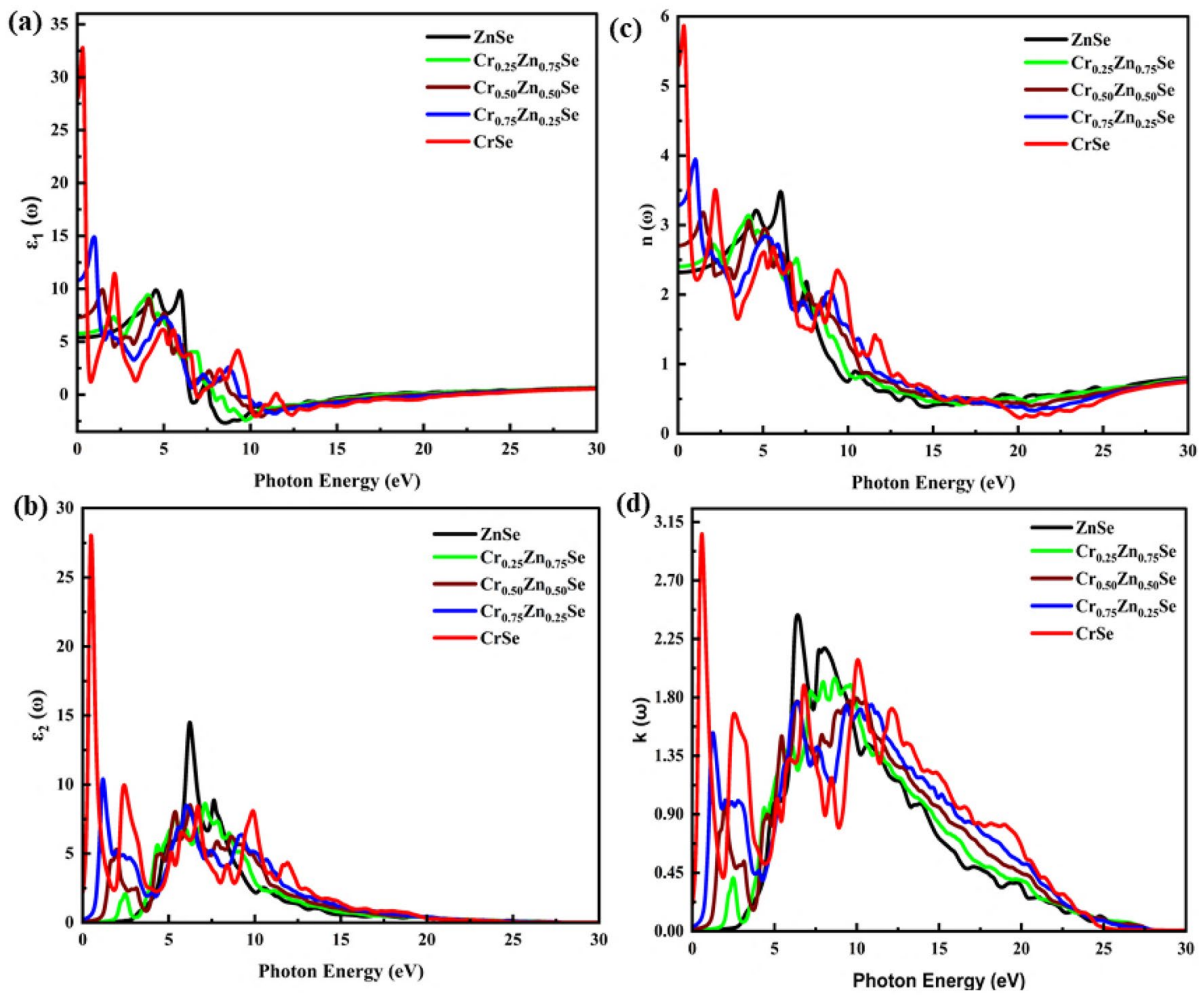
Change in the optical parameters at varying Cr-component (0, 0.25, 0.50, 0.75, 1). (**a**) ${\varepsilon }_{1}\left(\omega \right)$, (**b**) ${\varepsilon }_{2}\left(\omega \right)$, (**c**) *n* $\left(\omega \right)$, and (**d**) $k\left(\omega \right)$.

**Figure 5:**
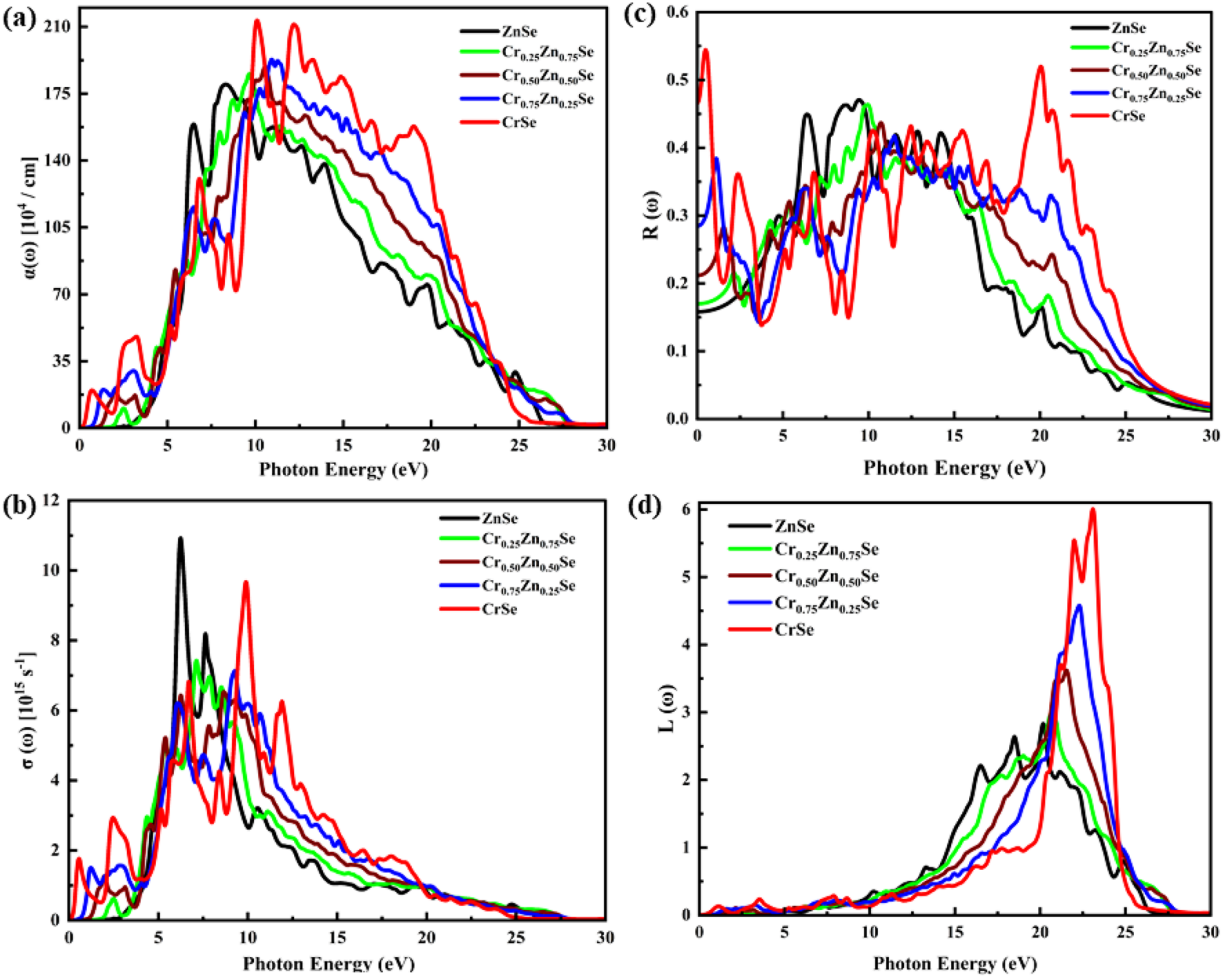
Optical parameters illustration at varying Cr concentration. (**a**) Optical absorbance, (**b**) Real part of conductivity, (**c**) Reflectivity, and (**d**) Energy loss function of interacting electrons.

The cubic symmetry depicts an isotropic optical response of ZnCrSe semiconductors. For further study of the optical characteristics, a denser mesh of k points was used to investigate the Cr content-dependent response of the materials to incident radiation with energy up to 30 eV. Cr introduces new peaks in the spectra of optical parameters (see Figs. 4 and 5). The real part of the dielectric function is shown in Fig. 4a, from which it can be inferred that the Cr originates a strong response in the infrared and visible radiation. This response is noticed to be strengthened as the Cr content is increased, depicting the active nature of these ZnCrSe materials. These materials show an active region corresponding to the energy of incoming light up to 10 eV. CrSe shows a more prominent response, mainly in the infrared and visible light ranges, and this response becomes stable after 12.50 eV. From these spectra, it can be deduced that the Cr content gives a boost to the dielectric response, depicting a strong response of these materials that spans mid-infrared-to-ultraviolet radiation having more pronounced transitions ranging from 0.22 to 7.25 eV^7^. The peak location is also observed to be shifted towards the visible range of wavelengths. The metallic behavior corresponding to the negative values of the spectra decreases with increasing Cr concentration, and all materials exhibit almost a static response above 15 eV, indicating a non-active region. The maximum peak and the static dielectric function’s (${\varepsilon }_{1}\left(0\right)$)^45^ highest values correspond to CrSe. The Cr content-dependent ${\varepsilon }_{1}\left(0\right)$ values increase with increasing Cr content, and the highest value is computed as 27.67 units, corresponding to CrSe while the minimum is 5.22 units, corresponding to ZnSe. The optical parameters value of these materials' response at an incident frequency of 0 Hz are summarized in Table 2. As the Cr content increases, the threshold energy of the imaginary part of the dielectric function keeps on decreasing, which raises the direct interband transitions at these points from the valence to the conduction band, depicting a downshift in the bandgap. This shift reduces the gap between the electrons of the valence and conduction bands and results in a change in the optical spectra (see Fig. 4b). Just like the real part of the dielectric function, these spectra are also dominated by CrSe, indicating that a high Cr content makes these materials more active in the infrared and visible range of light^7^. A pronounced shift can be seen in this figure, which depicts strong optical absorbance governed by ${\varepsilon }_{2}\left(\omega \right)$ depending on photon energy up to 5 eV with the incorporation of Cr. The peak height also shifted from 14.9 units (for ZnSe) to 27.85 units (for CrSe). In addition, for Cr content of 25, 50, and 75 percent, the spectra show a decrease in the peak height as compared with ZnSe. These materials show a static response to incoming radiation above 18 eV, showing inactivity for extreme ultraviolet light.

**Table 2:** A summary of Cr content-induced linear optical parameters.

Semiconductor	${\varepsilon }_{1} \left(0\right)$	*n* $\left(0\right)$	$R\, \left(0\right)$	${\rm E}$ (eV)	M (eV)^0.5^
ZnSe	5.224	2.285	0.153	2.546	0.357
Cr_0.25_Zn_0.75_Se	5.585	2.363	0.164	1.686	0.290
Cr_0.50_Zn_0.50_Se	7.090	2.663	0.206	1.018	0.226
Cr_0.75_Zn_0.25_Se	10.537	3.246	0.279	0.609	0.175
CrSe	27.677	5.265	0.464	0.141	0.084

The information related to the reduction in the speed of the incoming radiation is investigated by analyzing the refraction spectra, which depict a strong response with an increase in Cr content (see Fig. 4c). The more pronounced spectra still belong to CrSe, depicting that the maximum speed of the radiation is lost in this material, boosting the absorption in the visible range of light. The general trend of the spectra remains identical except for a prominent shift of peaks towards incident radiation with low photon energy. The main active region lies from 0 to 15 eV and a static response of these materials is observed above 26 eV of incoming radiation. The static refractive index (*n* $\left(0\right)$) of these materials experiences an increase with increasing Cr content and shifts from 2.285 (for ZnSe) to 5.265 units (for CrSe). Figure 4d depicts the change in the extinction coefficient ($k\left(\omega \right)$) as a function of Cr content, and from this figure, it can be seen that a strong response appears for CrSe. The peak height drops for 25, 50, and 75 percent of the Cr content. Also, a new region of optical spectra originated with the presence of Cr, depicting strong optical absorption in the infrared to the visible range of light. It is also observed that a steady spectrum is shown above 27.50 eV, which presents an ignorable absorption at these high energies.

The optical absorbance spectra of these materials with a changing Cr content are presented in Fig. 5a, of which variations are attributed to ${\varepsilon }_{2}\left(\omega \right)$ and $k\left(\omega \right)$. In general, the spectra are dominated by the ultraviolet range of light absorption, ranging from 10 to 28 eV. The presence of Cr induces a new absorbance spectrum in the visible and infrared ranges of light, which is not present for ZnSe^7^. These newly originated spectra depict a rise with an increase in Cr content, inferring the potential use of these alloyed materials for spintronics and optoelectronics. These materials show a wider spectrum of active regions, spanning up to 25 eV. The maximum absorption lies in the range of 6 eV to 25 eV, wherein ZnSe dominates the spectra up to 10.25 eV, while the Cr content-induced absorbance is significantly boosted in the range of 10.25 to 22.45 eV. The analysis of these spectra reveals that CrSe is prevalent and follows the same pattern as the extinction coefficient. Above the incident photon energy of 27.90 eV, a steady response of these materials is seen. The optical bandgap (${\rm E}$) and magnetization factor (M = $\sqrt{{({\rm E})}/20}$) are further determined by interpreting the absorption data using the Tauc’s plot method^46^ and are summarized in Table 2. The magnetization factor can be used to predict the nature of precipitated nanoparticles in a solid medium like glass–ceramics. A value of 1 suggests an insulating nature; a value closer to 0 implies metal-like behavior; and an intermediate between 0 and 1 represents a semiconducting nature. Here it is decreased significantly from 0.357 to 0.084, depicting a semiconducting to metallic nature. Figure 5b shows the real part of the optical conductance as a function of Cr content in the ZnCrSe materials, from which it can be noticed that visible and infrared ranges of light spectra originated, which increased with Cr content, leading to a new active region from 0 to 4 eV. The second active region ranges from 5 to 13 eV and is dominated by ZnSe following CrSe. The highest conductance value corresponds to ZnSe. For the CrSe spectrum, new peaks have been seen. In general, the peak location shifts to the incident radiation with high photon energy as the portion of Cr increases, while a steady spectrum is observed above 27.60 eV. The reflectance spectra also follow the same trend as the other optical parameters and the maximum reflectance corresponds to CrSe (see Fig. 5c). These spectra depict three active regions, showing a shift and an increase depending on the Cr content. The first region (from 0 to 4 eV) is dominated by CrSe, having new peaks, while the second region (from 5 to 12 eV) belongs to ZnSe. The third region is again dominated by CrSe and spans a wider range of 16 to 30 eV. The value of reflectance at zero energy (*R*(0)) increased sharply from 0.1531 to 0.4640 units subjected to the Cr concentrations. The energy loss spectrum is shown in Fig. 5d, from which it can be inferred that the maximum energy is lost in the region comprising 20 to 25 eV, and it belongs to CrSe. A shift of these spectra towards extreme ultraviolet light is also noticed. ZnSe has the minimum energy loss function. However, it is considerable only before the incoming photon energy of 27 eV, while it can be ignored under 0.25 eV with the main peak detection range of 15 eV to 25 eV with increasing Cr concentrations.

## Conclusions

This study investigated the Cr-based ZnSe semiconductors by utilizing the FP-LAPW method within the DFT and reports their cubic symmetry with a decrease in lattice constant as a function of Cr content. It is noticed that the substitution of Cr in the ZnSe lattice generates impurity bands and defects that significantly impact the electronic bandgap. A shift from direct to indirect bandgap nature is also seen at 50 and 75 percent content of Cr, along with boosted light absorption in the visible and infrared range. It demonstrates the potential of Cr doping in engineering ZnSe's electronic bandgap, presenting new device characteristics applicable in the optoelectronic realm, especially for innovative bandgap-utilizable devices.

### Supplementary Information

Supplementary Information.

## Data Availability

All data generated or analyzed during this study is included in this published article and supplementary material.
