# Supplementary material for: Investigating structural and optoelectronic properties of Cr-substituted ZnSe semiconductors
Source: Sci Rep. 2024 Jul 5;14:15510. doi: 10.1038/s41598-024-66378-2 (PMC11226455; doi:10.1038/s41598-024-66378-2)
Supplement: Supplementary file 1 — Supplementary Information. [file 41598_2024_66378_MOESM1_ESM.docx]

**Investigating Structural and Optoelectronic Properties of Cr-Substituted ZnSe Semiconductors**

Muhammad Aamir Iqbal^1,^*, Sunila Bakhsh^2^,  Siti Sarah Maidin^3^, Kareem Morsy^4^, Jeong Ryeol Choi^5,^*, Arnold C. Alguno^6,7^

1. School of Materials Science and Engineering, Zhejiang University, Hangzhou, 310027, China
2. Department of Physics, Balochistan University of Information Technology, Engineering and Management Sciences, Quetta 87300, Pakistan
3. Faculty of Data Science and Information Technology, INTI International University, Nilai 71800, Malaysia
4. Biology Department, College of Science, King Khalid University, Abha 61421, Saudi Arabia
5. School of Electronic Engineering, Kyonggi University, Suwon, Gyeonggi-do 16227, Republic of Korea
6. Department of Physics, MSU-Iligan Institute of Technology, Iligan City, 9200, Philippines
7. Research Centre for Energy Efficient Materials, Premier Research Institute of Science and Mathematics, MSU-Iligan Institute of Technology, Iligan City, 9200, Philippines

***Corresponding Authors Email:** [aamir.hum@gmail.com](mailto:aamir.hum@gmail.com) (MA Iqbal); [choiardor@hanmail.net](mailto:choiardor@hanmail.net) (JR Choi)


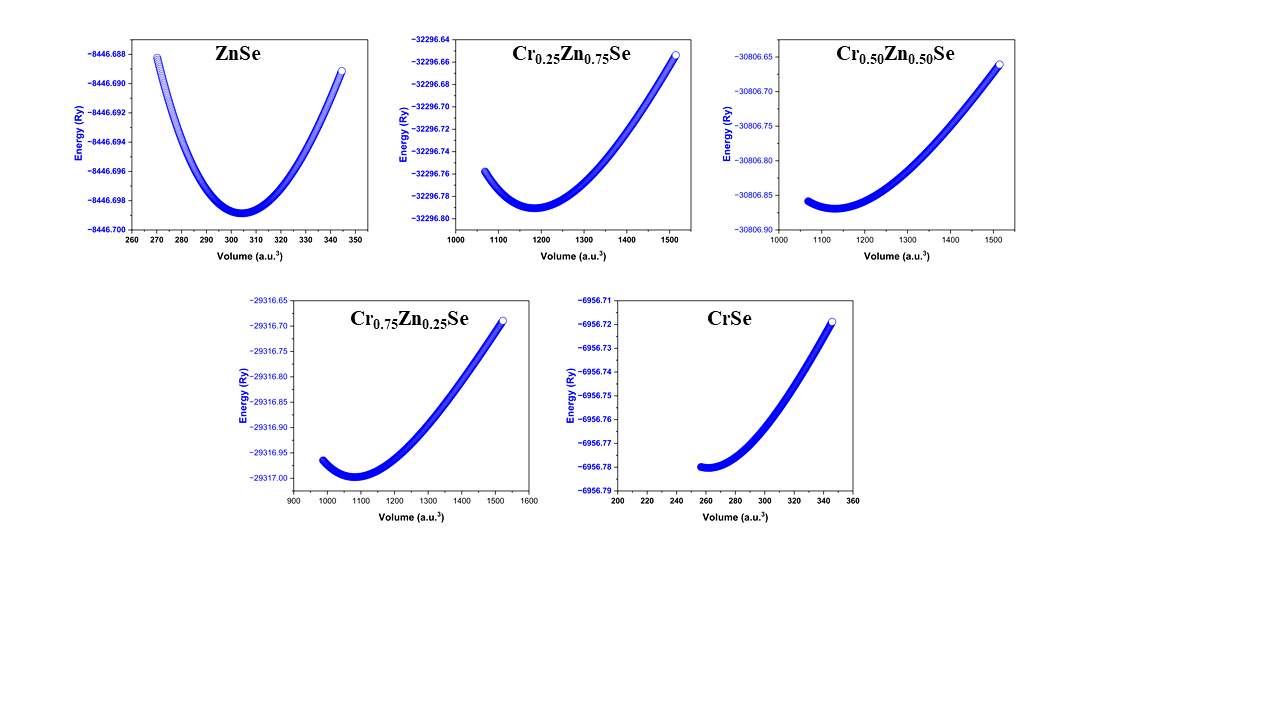


**Figure S1:** Optimization plots of minimum energy vs. ground state volume at varying Cr concentrations between 0 to 1.

**Table S1:** Cr content-dependent structural parameters determined within the Perdew-Burke-Ernzerhof generalized gradient approximation (GGA-PBE) functional.

| Semiconductor | Lattice Constant  (Å) | Bulk Modulus  (GPa) | Energy  (Ry) |
| --- | --- | --- | --- |
| ZnSe | 5.6496 | 66.6739 | -8446.698862 |
| Cr_0.25_Zn_0.75_Se | 5.5985 | 70.0603 | -32296.790567 |
| Cr_0.50_Zn_0.50_Se | 5.5128 | 82.6298 | -30806.869242 |
| Cr_0.75_Zn_0.25_Se | 5.4321 | 98.5056 | -29316.997638 |
| CrSe | 5.3724 | 113.8128 | -6956.780292 |


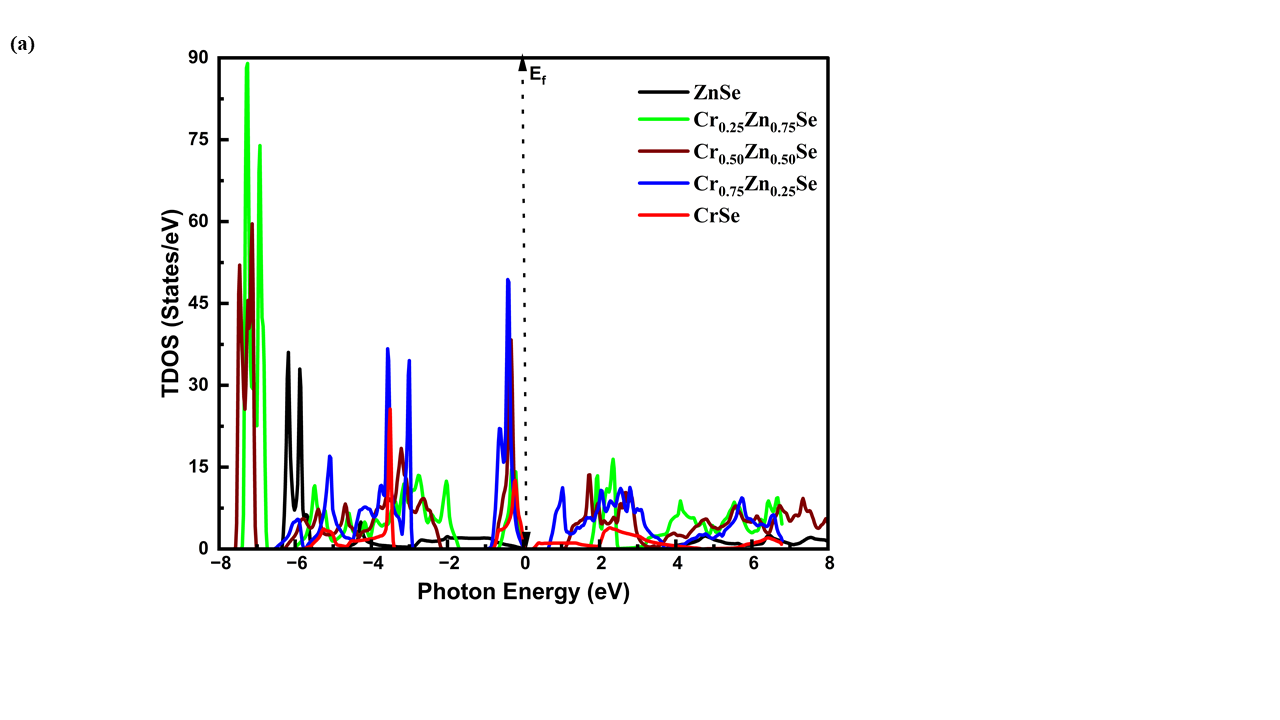


**Figure S2:** Change in TDOS of Zn_1-x_Cr_x_Se (x=0, 0.25, 0.50, 0.75, 1) semiconductors depending on incident photon energy between -8 to 8 eV.
